# Acrosyl

**Published:** 1914-09-19

**Authors:** 


### Institutional Needs.

### "ACROSYL."

A favourite type of antiseptic is the one in which mixtures of cresols are dissolved in liquid soap. There are several proprietary disinfectants of this type now enjoying a successful vogue in spite of some minor drawbacks. To this class belongs the new preparation which has been placed on the market under the name of "Acrosyl." In appearance this disinfectant has no particularly distinctive characteristics; it is a clear brown homogeneous liquid, with a pleasant smell. With water it mixes easily in all proportions. The strong solutions are perfectly clear and transparent, the weaker ones, such as the commonly used 1 per cent. strength, are cloudy, like those made with other members of this class of disinfectant. "Acrosyl" is very convenient for ordinary surgical work, and after practical trial is not found irritating in any way to the skin. This is in accordance with the claim made by the makers that "Acrosyl" is superior to other products of a similar nature because the cresols therein have been specially treated, and those irritant bodies which exert a caustic effect have been removed. This caustic effect has always been a drawback to preparations of this type, and as "Acrosyl" is prepared from purified ingredients and contains from 50 to 55 per cent. of the specially treated cresol in neutral soap, it will be found milder and less caustic. For gynæcological work it should undoubtedly find favour. The price compares favourably with those of its competitors, and it is said to be entirely of British manufacture. The makers are the Acrosyl Company, Sutherland House, Lloyd's Avenue, E.C.

